# Altered resting-state amplitudes of low-frequency fluctuations in offspring of parents with a diagnosis of bipolar disorder or major depressive disorder

**DOI:** 10.1371/journal.pone.0316330

**Published:** 2025-02-18

**Authors:** Mélanie Boisvert, Jules R. Dugré, Stéphane Potvin

**Affiliations:** 1 Research Center of the Institut Universitaire en Santé Mentale de Montréal, Montreal, Canada; 2 Faculty of Medicine, Department of Psychiatry and Addictology, University of Montreal; Montreal, Canada; 3 Centre for Human Brain Health & School of Psychology, University of Birmingham, Birmingham, United Kingdom; University of North Carolina at Chapel Hill, UNITED STATES OF AMERICA

## Abstract

Offspring of parents with bipolar disorder (BD) or major depressive disorder (MDD) are at high biological risk (HR) of these disorders given their significant heritability. Thus, studying neural correlates in youths at HR-MDD and HR-BD appears essential to understand the development of mood disorders before their onset. Resting-state amplitudes of low-frequency fluctuations (ALFF) and fractioned ALFF (fALFF) shows moderate to high test-retest reliability which makes it a great tool to identify biomarkers. However, this avenue is still largely unexplored. Using the *Healthy Brain Network biobank*, we identified 150 children and adolescents HR-MDD, 50 HR-BD and 150 not at risk of any psychiatric disorder (i.e., the control group). We then examined differences in relative ALFF/fALFF signals during resting-state. At a corrected threshold, participants HR-MDD displayed lower resting-state ALFF signals in the dorsal caudate nucleus compared to the control group. The HR-BD group showed increased fALFF values in the primary motor cortex compared to the control group. Therefore, robust differences were noted in regions that could be linked to important symptoms of mood disorders, namely psychomotor retardation, and agitation. At an uncorrected threshold, differences were noted in the central opercular cortex and the cerebellar. The database is a community-referred cohort and heterogeneous in terms of children’s psychiatric diagnosis and symptomatology, which may have altered the results. ALFF and fALFF results for the comparison between both HR groups and the control group overlapped, suggesting good convergence. More studies measuring ALFF/fALFF in HR are needed to replicate these results.

## Introduction

Bipolar disorder (BD) and major depressive disorder (MDD) are leading causes of morbidity [1] and highly associated with suicide [2,3]. BD is a cyclic and chronic disorder with a lifetime prevalence of 2.4% [4] and first emerges in the majority as a depressive episode in late adolescence or early adulthood [5]. MDD has a lifetime prevalence of around 11% in individual of 13 years and older [6,7] and is in most cases episodic. Distinguishing BD from MDD in a young patient can be challenging but is crucial since poor management of BD can worsen prognosis and have significant consequences for the individual, such as increased hospitalization, suicide attempts and treatment resistance [8,9]. To this day, mood disorders are diagnosed based on self-reported symptoms. Still, BD cannot be distinguished from MDD with self-report data only, unless a (hypo)manic episode has occurred which highlights the need to discover other markers. Given that BD has a strong genetic component (≤85% of heritability) [10,11] and that MDD also has a significant heritability (≤44%) [12,13], studying neurobiological mechanisms appears important. Likewise, as mood disorders can be hereditary, examining neurobiological processes in young offspring of patients with BD or MDD can give us better insight on biomarkers prior to the onset of the disorder.

Studying brain activity and connectivity represents an important tool for understanding the mechanisms that underlies symptoms in psychiatric disorders, and therefore better characterize the pathophysiology of the disorder. For example, it has been shown that altered activity of the brain reward system (e.g., striatum, ventrolateral prefrontal cortex, amygdala) is linked to hypersensitivity to reward in BD [14]. On the other hand, hypoactivation of this system is associated with depressive episodes and anhedonia in both BD and MDD [15]. Notably, a study in preadolescents of parents with a diagnosis of either MDD or BD, who are therefore at biological high-risk (HR) of mood disorders, highlighted aberrant activation of the putamen, ventrolateral prefrontal cortex and cerebellar during reward outcome processing in comparison to offspring of healthy parents [16]. With the aim to identify differences between the risk of BD vs MDD, the authors compared the two HR groups and found that aberrant connectivity and reduced activity of the thalamic during reward processing distinguished the HR-BD group from the HR-MDD group. Importantly, a similar pattern was also noted in patients with a diagnosis of BD compared to patient with a diagnosis of MDD during a reward condition [17]. Mood disorders are also associated with emotion dysregulation which is supported by the common increased engagement of limbic regions during emotional processing tasks observed in both disorders [18]. Echoing these results, a few studies using emotional processing tasks and regions of interest analyses found increased activations of limbic regions in participants HR-BD or HR-MDD [19–22], while others did not [23–25]. Moreover, studies examining resting-state brain dysconnectivity highlighted alterations of the limbic neurocircuit in BD both in patients and their first-degree relatives [26,27].

Studying the brain activity at rest allows the identification of aberrant spontaneous activity and connectivity during task-free cognitive and self-referential states of mind (e.g., mind wandering). Brain activity measured by amplitudes of low-frequency fluctuations (ALFF) shows moderate to high test-retest reliability [28]. Several studies have examined ALFF in mood disorders [29]. A meta-analysis of this literature highlighted common altered brain activity in the insula, the medial prefrontal cortex and cerebellum based on 50 MDD studies and 15 BD studies. Differences between MDD and BD were also noted in several regions such as the striatum, the anterior and posterior cingulate cortex [29]. Examination of fALFF signals in siblings of patients with MDD has highlighted alterations in the dorsomedial prefrontal cortex [30]. A study focusing on resting-state ALFF in mid-to-late teenagers found decrease ALFF in the left putamen in Chinese healthy offspring of patients with BD (n = 28) in comparison to controls. The authors also included a group of offspring of patients with BD that presented subthreshold symptoms of BD (n = 22) which showed increased ALFF in right cerebellum in comparison to the control group [31]. Finally, one study done in New-York (USA) focused on ALFF signals and brain connectivity in adults at genetic risk of MDD (n = 44) (i.e., children or grandchildren of patients with MDD). In their exploratory whole-brain analyses, the authors found that a family history of MDD was associated with decreased ALFF signal in the posterior cingulate cortex and increased fractioned ALFF (fALFF) in the dorsomedial prefrontal cortex [32]. To our knowledge, these last two studies are the only ones examining resting-state ALFF in *offspring* of patients with BD or MDD which highlights the inexistant literature in young cohorts such as late childhood or early adolescence. Given the scarcity of resting-state neuroimaging studies examining ALFF and fALFF signals in youth at HR-BD and HR-MDD, investigating the common and distinct brain features are utmost importance.

Therefore, the aim of the present study was to compare whole-brain resting-state ALFF and fALFF signals of young offspring of patients with either a BD or MDD and offspring of healthy parents using the *Healthy Brain Network* (HBN) biobank. To the best of our knowledge, no neuroimaging studies in offspring have examined resting-state ALFF and fALFF in a younger cohort such as late childhood and early adolescence. The present study is expected to advance knowledge on ALFF and fALFF alterations in HR-BD and HR-MDD for this developmental period specifically. Additionally, this study may contribute to a better characterization of the potential neurobiological mechanisms underlying the risk for BD and MDD.

## Methods

### Participants

Using the HBN biobank Data Release 7.0, data from 2,200 participants were obtained on October 21st, 2019. HBN is an initiative from the Child Mind Institute that adopted a community-referred recruitment model to investigate developmental psychopathologies in children and adolescents (5–21 years) in New York City area (N.Y., USA). To capture the heterogeneity in psychopathologies, exclusion criteria only concerned the presence of impairments that prevents full participation in the study (e.g., hearing or visual impairments, IQ below 66, being non-verbal, etc.), recent onset of serious psychiatric disorder (e.g., psychotic episode, etc.) or suicidality/homicidality. Other exclusion criteria were acute encephalopathy, acute substance intoxication, and neurodegenerative disorder. Prior to enrolling in the study, written assent was obtained from participants younger than 18 years old, and written informed consent was obtained from their legal guardians. Written consent was obtained from participants aged 18 or older. More details are provided elsewhere [33]. The original HBN study was approved by the Chesapeake institutional review Board (https://www.chesapeakeirb.com/) and the current study was approved by the local ethics committee. The investigation was carried out in accordance with the latest version of the Declaration of Helsinki.

From the 2,200 participants, 1,583 had available functional neuroimaging data and from these, 1,416 participants had valid resting-state functional magnetic resonance imaging (fMRI) data (see section 2.3). Using data from the Family History–Research Domain Criteria (FH-RDC) [34] (see section 2.2.1.) we aimed to identify offspring at genetic risk of BD and MDD. For the HR-BD group, the participants had to have at least one parent with a diagnosis of BD type I or type II. HR-BD that also had a parent with a past or present diagnosis of MDD were excluded. For the HR-MDD group, the participants had to have at least one parent with a diagnosis of past or present MDD or dysthymia. HR-MDD that also had a parent with a diagnosis of BD were excluded. Finally, the control group was composed of participants which neither of their parents that had a past or present psychiatric diagnosis. From the 1,416 participants, 50 were identified as HR-BD, 181 as HR-MDD and 675 were not at risk of any psychiatric disorder. The three groups did differ on the scanner used (e.g., study site; see section 2.3; χ^2^(4,906) = 12.912, p = 0.012). Groups also differed on the full-scale intellectual quotient (FSIQ) composite score (F(2,906) = 5.55, p = 0.004) as measured by the Wechsler Intelligence Scale for Children (WISC) [35]. Therefore, the three groups were matched for age, sex, study site and FSIQ composite score using the MatchIt package on R software (version 4.2.2.). It was decided to match the HR-MDD group and the control group to the HR-BD group to keep the full 50 HR-BD sample. A ratio of 1:3 was selected to match the three groups very closely.

### Clinical variables

#### Biological risk identification

The HBN biobank includes a collection of psychiatric, cognitive, and behavioral assessments. First, the genetic risk factor was assessed using the FH-RDC, as introduced above, which is an instrument that opts to collect information from the patient or a relative about the family history through an anamnesis [34]. The FH-RDC has specific diagnostic criteria that allows to make a diagnosis, if needed, based on the interview. The sensitivity of the FH-RDC to accurately diagnose any psychiatric disorder in family members has been estimated at 57% for mood disorders, while the specificity has been evaluated at 93% for mood disorders [36].

#### Psychiatric disorders of offspring

To establish psychiatric diagnoses of participants (i.e., offspring), a licensed clinician administered parent interview and child interview using the computerized version of the Kiddie Schedule for Affective Disorders and Schizophrenia-Children’s version (KSADS-COMP) [37]. The KSADS-COMP is a well validated semi-structured diagnostic interview that provides automated diagnoses. The HBN clinical team established psychiatric diagnoses by clinical consensus based on results of the KSADS-COMP and other data provided during participation.

#### Sociodemographic and psychometric measures

Socio-demographic information (i.e., age, race, and sex of the participant and annual household income, etc.) was acquired through parent-report and intellectual quotient was assessed using the FSIQ from the WISC. Handedness was evaluated using the Edinburgh Handedness Questionnaire (EHQ) which provides laterality index scores ranging from -100 (i.e., left-handed) to 100 (i.e., right-handed), with 0 meaning ambidextrous. EHQ scores were converted in nominal 0,1,2 for left-handed (score<-40), ambidextrous (i.e., score between -40 and 40), and right-handed (score>40) [38]. Considering the high prevalence of anxio-depressive symptoms among young people at risk of mood disorders [39], total scores were derived from the Screen for Child Anxiety Related Disorders–Parent Report (SCARED-P) [40] and the Mood and Feelings Questionnaire–Parent Report (MFQ-P) [41]. The SCARED is composed 41-items of 3-point scale (0 = not true or hardly ever true; 1 = somewhat true or sometimes true; 2 = very true or often true) and a total score of ≥25 may indicate the presence of an anxiety disorder. This scale showed good internal consistency (α = 0.929). The MFQ-P is a 34-items screening tool for depression in youth. This scale opts to capture how the child has been feeling the past two weeks with descriptive phrases that are rated as either 0 = not true, 1 = sometimes true, or 2 = true. Internal consistency of the MFQ-P was good (α = 0.929). Severity of attention deficit disorder with hyperactivity (ADHD) symptoms were assessed using the average score of the Strengths and Weaknesses Assessment for ADHD and Normal behavior (SWAN) [42]. The SWAN is an 18-items rating scale that evaluates problem behavior, hyperactivity, and attention deficits, with scores ranging from -3 (i.e., strength) to 3 (i.e., weakness). In this study, the internal consistency of the SWAN scale was considered good (α = 0.956). Finally, the total number of negative events experienced as assed from the 21-items Negative Life Events Scale–Parent Report (NLES-P) [43].

### fMRI image acquisition and preprocessing

MRI acquisition was done at three different sites: (1) mobile 1.5T Siemens Avanto in Staten Island, (2) 3T Siemens Tim Trio at Rugers University Brain Imaging Center (RUBIC), and (3) 3T Siemens Prisma at the CitiGroup Ornell Brain Imaging Center (CBIC). Data for the mobile site in Staten Island were acquired in a single run lasting 10 minutes, while both RUBIC and CBIC data acquisition protocols consisted of two resting-state scans lasting 5min each. Importantly, the vast majority of participants (90%) came from site 2 (53%) and site 3 (37%), which used the same acquisition parameters on 3T scanners, while only a small minority came from site 1 (1.5T, 10%). Considering that scanner differences (1.5T vs 3T) are not well captured by Combat [44], we decided to match groups on study site instead of using ComBat. For all resting-state functional runs, participants viewed a fixation cross located at the center of the computer screen. More details are available in Supplementary Methods.

MRI data was collected by the study team of HBN and was processed by our team [45,46]. First, functional images were realigned, corrected for motion artifacts with the Artifact Detection Tool [47] (ART, setting a threshold of 0.9mm subject ART’s composite motion and a global signal threshold of Z = 5) with the implemented in CONN Toolbox [48], and co-registered to the corresponding anatomical image. Then, the anatomical images were segmented (into grey matter, white matter, and cerebrospinal fluid) and normalized to the Montreal Neurological Institute (MNI) stereotaxic space. Functional images were then normalized based on structural data, spatially smoothed with a 6mm full-width-at-half-maximum 3D isotropic Gaussian kernel and resampled to 2mm^3^ voxels. For the preprocessing, the anatomical component-based noise correction method (aCompCor strategy, [49]), was employed to remove confounding effects from the BOLD time series, such as the physiological noise originating from the white matter and cerebrospinal fluid. This method was found to increase the validity and sensitivity of analyses [50]. The time-series were band-pass filtered (0.01Hz<f<0.10Hz). Images were manually checked for each of the 1,583 participants. We found pre-processing issues due to the poor quality of images in 108 participants, which resulted in the software being unable to adequately detect and segment volumes into tissue classes (i.e., grey matter, white matter, and cerebrospinal fluid). In addition, 59 adolescents exhibited high movements (exceeding 3 mm) leaving a remaining sample of 1,416 participants.

Brain activity was assessed by ALFF and fALFF. ALFF assess the strength of spontaneous neural activity within the low frequency band at rest [51] and can be calculated as the rood mean square of BOLD signal at each individual voxel after band-pass filtering [52]. ALFF values were divided by the whole brain mean ALFF values to normalize the global effects and fALFF was scaled by the total power relative to the full frequency range [53].

### fMRI between-group comparison analysis

Second level analysis was done using SPM12 (Statistical Parametric Mapping, UK) software package based on Matlab R2022b (version 9.13.0, MathWorks Inc., MA). Our principal analysis was an analysis of variance (F-test) to compare ALFF and fALFF values between the three groups with percentage of valid fMRI scans and mean head motion as covariates. We also compared the combined HR groups with the control group. Finally, we carried out exploratory analyses directly comparing the HR groups with each other and each with the control group. For statistical significance, we used a voxel-forming threshold of p<0.001, a cluster-level threshold of p<0.05 and a cluster extend of k≥20 voxels [54]. Then we performed correction for multiple comparison by false discovery rate (FDR) with a threshold of p_FDR_<0.05. Relative ALFF and fALFF values of significant uncorrected clusters from the F-tests were extracted using MarsBar, and implemented in SPSS (version 28, SPSS Inc., Chicago, IL, USA). Pairwise comparisons were defined as significant at a threshold of p<0.01 Bonferroni corrected. Analyses of covariance were performed to examine whether the results were driven by differences between groups in severity of potentially confounding clinical symptoms. Sensitivity analyses were performed by calculating the effect size for each impaired region for each group comparison with and without the study site 1.

### Mental functions and neurochemical correlates

To obtain mental functions associated with the clusters, meta-analytic coactivation modelling [55] was conducted on the significant results obtained via the F-tests and was performed using NeuroQuery Database [56] which include 13,459 studies. For each of our results, voxels of the unthresholded meta-analytic coactivation map were correlated via pearson correlation with those from 13 Data-Driven Maps derived from 1,347 task-based fMRI meta-analyses [57]. Also derived from these maps are 19 receptor/transporter density maps spanning 9 different neurotransmitter systems [58] including serotonin (i.e., 5-HT_1A_, 5-HT_1B_, 5-HT_2A_, 5-HT_4_, 5-HT_6_, 5-HTT), dopamine (i.e., D_1_, D_2_, DAT), norepinephrine (i.e., NET), histamine (i.e., H_3_), acetylcholine (i.e., α4β2, VAChT), cannabinoid (i.e., CB_1_), opioid (i.e., MOR), glutamate (i.e., NMDA, mGluR_5_) and GABA (i.e., GABA_A/BZ_). The top three most correlated functions and neurotransmitter maps are reported, and other results with moderate to strong correlation (r≥0.30) are detailed in Supplementary Material.

## Results

### Demographic and clinical variables

The HR-MDD, HR-BD and control group did not differ in terms of age, sex, FSIQ, and study site (Table 1). However, the HR-MDD group was composed of fewer Black/African American participants in comparison to the HR-BD group. Groups did not differ in terms of handedness and U.S household income classification. For the clinical data, groups did not differ in terms of diagnosis (all p-values>0.1, Table 2). HR-MDD had a higher mean average score on the SWAN compared to the control group (F(2,330) = 4.459, p = 0.024). Both HR-MDD and HR-BD had a higher number of negative life events compared to the control group (F(2,316) = 10.363, p<0.001 and p = 0.008 respectively), but did not differ from each other. Finally, mean head motion and percentage of valid scans did not differed between groups (F(2,350) = 0.998, p = 0.370 and F(2,350) = 0.768, p = 0.465 respectively, S1 Table). However, fALFF data from one participant of the HR-MDD group was excluded due to poor data quality.

**Table 1:** Participants demographic.

	HR-MDD group (n = 150)	HR-BD group (n = 50)	Control group (n = 150)	Analysis	
	**Mean (sd)**			**Statistic**	**p-value**
**Age**	11.38 (3.43)	11.56 (3.34)	11.66 (3.64)	F(2, 350) = 0.246	0.782
**WISC** ^a^	96.79 (13.59)	92.61 (13.80)	93.90 (16.05)	F(2, 350) = 2.182	0.114
	**n (%)**			**Statistic**	**p-value**
**Female**	60 (40.0%)	23 (46.0%)	75 (50.0%)	χ^2^(2, 350) = 3.046	0.218
**Handedness**					
Left-handed	3 (2.0%)	5 (10.0%)	8 (5.3%)	χ^2^(4, 344) = 5.957	0.202
Ambidextrous	20 (13.3%)	7 (14.0%)	19 (12.7%)		
Right-handed	123 (82.0%)	37 (74.0%)	122 (81.3%)		
**Race**					
White/Caucasian	67 (44.7%)	12 (26.0%)	50 (33.3%)	χ^2^(8, 277) = 19.455	**0.013** *
Black/African American	10 (6.7%)	11 (22.0%)	22 (14.7%)		
Hispanic	22 (14.7%)	5 (10.0%)	14 (9.3%)		
Asian	2 (1.3%)	1 (2.0%)	8 (5.3%)		
Two+ races, or other race	20 (13.3%)	9 (18.0%)	23 (15.4%)		
**U.S. household income class**					
Low-income (< 50k/year)	33 (24.3%)	12 (27.9%)	24 (18.0%)	χ^2^(6, 312) = 7.875	0.247
Middle-income (50k-150k/year)	51 (37.5%)	18 (41.9%)	55 (41.4%)		
High-income (≥ 150k/year)	39 (28.7%)	6 (14.0%)	32 (24.1%)		
Chose not to disclose	13 (9.6%)	7 (16.3%)	22 (16.5%)		
**Study site**					
Staten Island RUBIC CBIC	21 (14.0%) 82 (54.7%) 47 (31.3%)	12 (24.0%) 26 (52.0%) 12 (24.0%)	33 (22.0%) 84 (56.0%) 33 (22.0%)	χ^2^(4, 350) = 6.076	0.194

**Note**.

*Significant with a p<0.05 threshold. HR-MDD = high-risk of major depressive disorder; HR-BD = high-risk bipolar disorder; WISC = Wechsler Intelligence Scale for Children

^a^ intellectual quotient composite score. Handedness information was not available for 4 HR-MDD, 1 HR-BD, and 2 controls. Data on annual household income was not available for 14 HR-MDD, 7 HR-BD, and 17 controls.

**Table 2:** Participants clinical data.

	HR-MDD group (n = 150)	HR-BD group (n = 50)	Control group (n = 150)	Analysis	
	n (%)			Statistic	p-value
**Diagnostic**					
No diagnosis	11 (7.3%)	5 (8.0%)	19 (12.7%)	χ^2^(2, 331) = 2.798	0.247
ADD/ADHD	80 (53.3%)	30 (60.0%)	74 (49.3%)	χ^2^(2, 331) = 2.798	0.247
Autism spectrum disorder	20 (13.3%)	3 (6.7%)	23 (15.3%)	χ^2^(2, 331) = 2.655	0.265
Other NDD	40 (26.7%)	11 (22.0%)	48 (32.0%)	χ^2^(2, 331) = 2.163	0.339
Depressive disorders	22 (14.7%)	4 (8.0%)	17 (11.3%)	χ^2^(2, 331) = 1.389	0.499
Conduct disorders	9 (6.0%)	6 (12.0%)	11 (7.3%)	χ^2^(2, 331) = 2.411	0.300
Anxiety disorders	43 (28.7%)	13 (26.0%)	38 (25.3%)	χ^2^(2, 331) = 0.263	0.877
Trauma disorders	5 (3.3%)	3 (6.0%)	3 (2.0%)	χ^2^(2, 331) = 2.200	0.333
Other disorders	10 (6.7%)	3 (6.0%)	8 (5.3%)	χ^2^(2, 331) = 0.189	0.910
	**Mean (sd)**			**Statistic**	**p-value**
**MFQ-P Total score**	11.11 (9.58)	9.47 (7.68)	8.47 (9.40)	F(2, 317) = 2.796	0.063
**SCARED-P Total score**	17.84 (13.28)	14.51 (10.17)	14.45 (12.27)	F(2, 316) = 2.870	0.058
**SWAN Average score**	0.59 (1.00)	0.65 (0.98)	0.25 (1.18)	F(2, 330) = 4.459	**0.012** *
**NLES-P**					
Total events	7.51 (3.33)	7.52 (3.86)	5.79 (3.20)	F(2, 322) = 10.363	**< 0.001** *

**Note**.

*Significant with a p<0.05 threshold. HR-MDD = high-risk of major depressive disorder; HR-BD = high-risk bipolar disorder; ADD = attention deficit disorder; ADHD = attention deficit hyperactivity disorder; NDD = neurodevelopmental disorder; MFQ-P = Mood and Feelings Questionnaire—Parent Report; SCARED-P = Screen for Child Anxiety and Related Disorders—Parent report; SWAN = Strengths and Weaknesses Assessment for ADHD and Normal behavior; NLES-P = Negative Life Events Scale—Parent Report. 5 HR-MDD, 5 HR-BD, and 9 controls dropped out of the study before a diagnosis was given.

### ALFF fMRI results

F-tests revealed between-group differences in amplitude of the dorsal caudate nucleus (Table 3 and Fig 1.1). Direct comparison between HR-MDD and controls revealed significant effect in the dorsal caudate (MNI = 16,16,22; 176 voxels) which survived FDR correction a the cluster level (p_FDR_ = 0.010) (S2 Table). In post hoc tests, a trend was also noted for lower ALFF values in the caudate cluster in the HR-MDD compared to the HR-BD group (p = 0.017). Functional decoding revealed that this cluster was associated with several mental functions such as motivation (r = 0.52), cognitive control (r = 0.52) and multi demand (r = 0.50). This cluster showed associations with several receptor/transporter density maps, such as D_2_ receptors (r = 0.51), D_1_ receptors (r = 0.51), and VAChT (r = 0.47) (S3 and S4 Tables).

**Fig 1:**
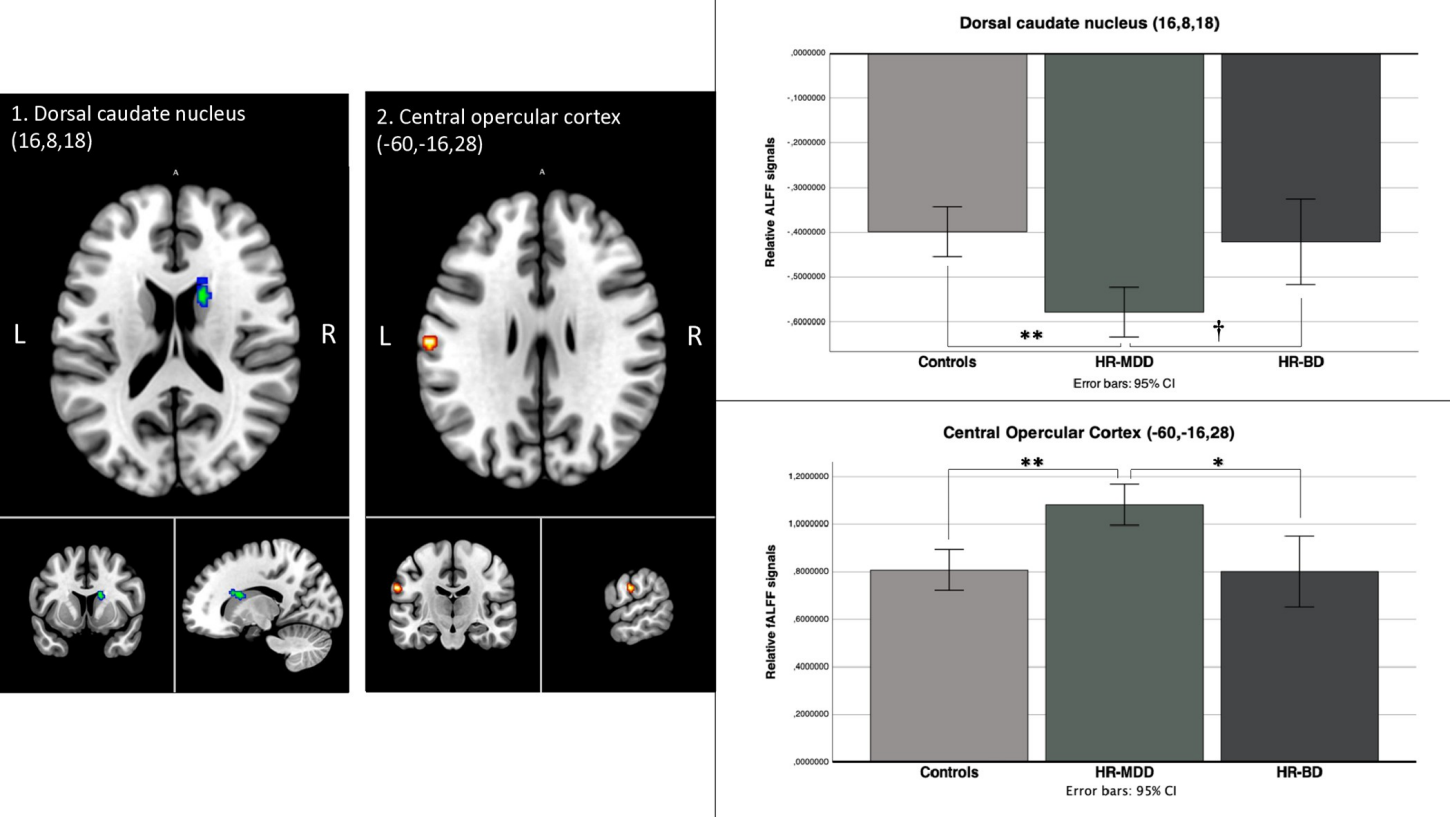
Clusters exhibiting lower relative ALFF (1) or fALFF (2) signals in HR-MDD compared to the control group when searching for any effect. Note. L = left; R = right; HR-MDD = participants at high-risk of major depressive disorder; ALFF = amplitude of low-frequency fluctuations; fALFF = fractioned amplitude of low-frequency fluctuations; * = significant at p<0.01 Bonferroni corrected; ** = significant at p<0.001 Bonferroni corrected; † = trend (p = 0.017).

**Table 3:** ALFF results.

	L/R	Regions	MNI coordinates (x,y,z)	Voxels	p_FDR_ (cluster-level)	F-value	p-value uncorrected (peak-level)
**ANY EFFECT**							
	**R**	**Dorsal caudate nucleus**	**16,8,18** **16,16,22**	**33**	**0.717**	**10.66**	**< 0.001**
**HR > CTRL**							
	R	Cerebellar, lobule VI	34,-44,-30 30,-36,-26	22	0.681	17.85	< 0.001
	R	Cerebellar, lobule VIII and VIIB	34,-60,-44	34	0.681	17.31	< 0.001
	L	M1	-38,-18,56 -34,-20,46	24	0.681	14.30	< 0.001

Note. Bold = Significant at a false-discovery rate (FDR) corrected threshold of p<0.05. ALFF = amplitudes of low-frequency fluctuations; HR-MDD = high-risk of major depressive disorder; HR-BD = high risk of bipolar disorder; MNI = Montreal Neurological Institute; M1 = primary motor cortex. Results in bold survived FDR correction for the direct comparison between HR-MDD and controls.

When comparing the combined HR groups with the control group (p<0.001, 20 voxels), results suggested the HR group had higher relative ALFF in a cluster located in the lobule VI of the cerebellar (Fig 2.1), another in the lobule VIII and overlapping with the lobule VIIB of the cerebellar (Fig 2.2) and in the primary motor cortex (M1) (Fig 2.3) compared to the control group (Table 3). No regions showed higher ALFF values in the control group relative to the HR group.

**Fig 2:**
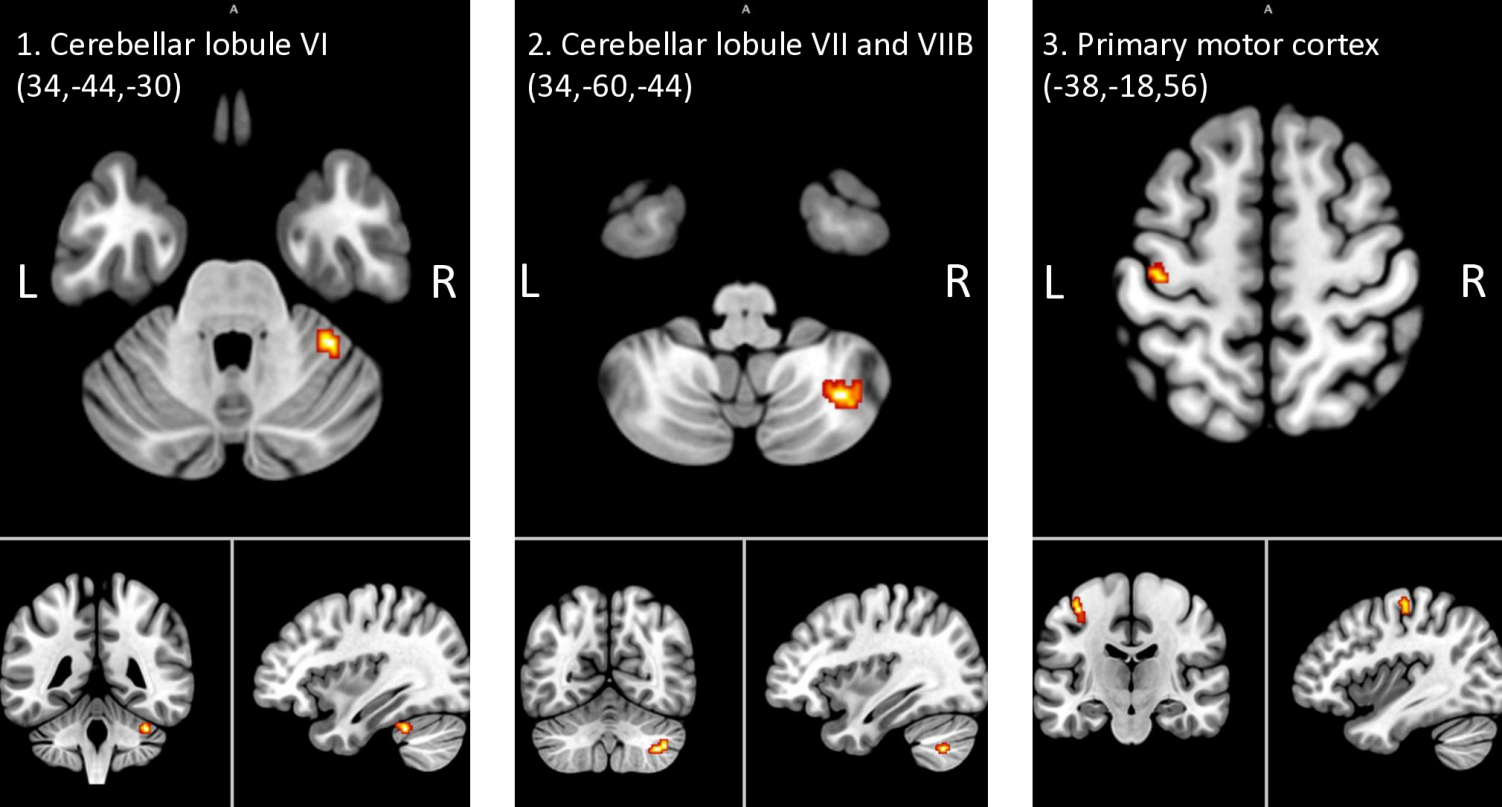
Clusters exhibiting higher relative ALFF signals in both HR groups compared to the control group (p<0.001). Note. L = left; R = right; HR = participants at high-risk of major depressive disorder or bipolar disorder; ALFF = amplitude of low-frequency fluctuations; fALFF = fractioned amplitude of low-frequency fluctuations.

When controlling for the SWAN average score, only the difference between the HR-BD group and the control group for the cerebellum lobule VIII and VIIB became a trend (p = 0.012), while the other group differences remained significant (S5 Table). When controlling for the total number of negative life events, both between-group differences for the cerebellar lobule VIII and VIIB were trends (p = 0.020 and p = 0.017 for the comparison between control group and HR-MDD or HR-BD, respectively).

Sensitivity analyses showed very minimal effects of study site 1 on results, based on the calculation of the effect sizes for each impaired region (S6 Table).

We also conducted exploratory whole-brain pairwise differences between HR-MDD and HR-BD which revealed no significant results (p<0.001, 20 voxels). Comparing HR-BD and controls yielded differences in the cerebellar lobule VI and the M1. Finally, comparing HR-MDD and controls highlighted differences in ALFF values in the posterior midcingulate cortex, dorsal cingulum bundle, midcingulate cortex, caudate, cerebellar lobule VIII and VIIB, inferior frontal gyrus and central opercular cortex (S2 Table).

### fALFF fMRI results

Group comparisons at a whole-brain level indicated difference in fALFF of the central opercular cortex (Table 4, Fig 1.2). Post-hoc test revealed the HR-MDD group showed higher relative fALFF in this cluster in comparison to the HR-BD and the control group (p = 0.004 and p<0.001, respectively). Functional decoding of this cluster showed association with physiological arousal (r = 0.56), action (r = 0.51), and cognitive control (r = 0.44). The cluster located in the central opercular cortex was associated with mGluR5 (r = 0.41), 5-HT_1B_ (r = 0.40), and NET (r = 0.39) (S3 and S4 Tables).

**Table 4:** fALFF results.

Contrast	L/R	Regions	MNI coordinates (x,y,z)	Voxels	p_FDR_ (cluster-level)	F-value	p-value uncorrected (peak-level)
**Any effect**							
	L	Central opercular cortex	-60,-16,28	20	0.596	10.85	< 0.001
**HR > CTRL**							
	R	Cerebellar, lobule VIII and VIIB	34,-62,-44 40,-66,-48	29	0.260	18.88	< 0.001
	**L**	**M1**	**-38,-18,54**	**26**	**0.260**	**16.82**	**< 0.001**

Note. Bold = Significant at a false-discovery rate (FDR) corrected threshold of p<0.05. fALFF = fractioned amplitudes of low-frequency fluctuations; HR-MDD = high-risk of major depressive disorder; HR-BD = high risk of bipolar disorder; MNI = Montreal Neurological Institute; Results in bold survived FDR correction for the direct comparison between HR-BD and controls.

When comparing the combined HR groups with the control group (p<0.001, 20 voxels), the HR groups showed increased fALFF signals in the cerebellar lobule VIII and VIIB (Fig 3.1) and M1 (Fig 3.2), while no regions showed higher relative fALFF in the control group (Table 4). Controlling for the number of negative life events and the average score on the SWAN did not affect the results (S5 Table).

**Fig 3:**
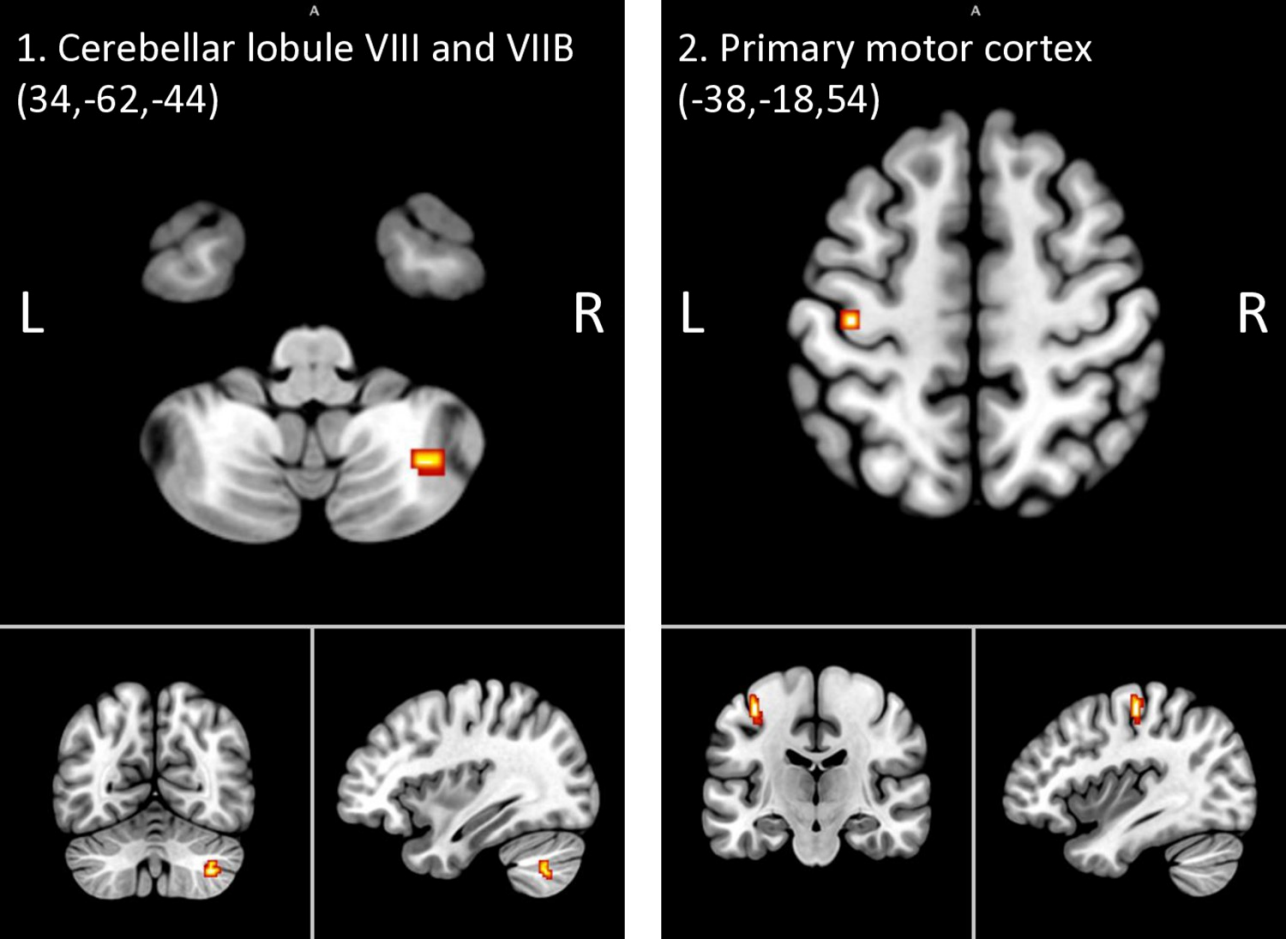
Clusters exhibiting higher relative fALFF signals in both HR groups compared to the control group (p<0.001). Note. L = left; R = right; HR = participants at high-risk of major depressive disorder or bipolar disorder; ALFF = amplitude of low-frequency fluctuations; fALFF = fractioned amplitude of low-frequency fluctuations.

Exploratory whole-brain pairwise differences between HR-MDD and HR-BD were found in clusters located in the inferior frontal gyrus and the lateral orbitofrontal cortex. Similarly, comparing HR-BD and controls yielded differences in the M1 and the cerebellar lobule VI. Importantly, the M1 result (MNI = -38,-18,52; 60 voxels) survived FDR correction (p_FDR_ = 0.042) (S7 Table). Comparing HR-MDD and controls showed differences in clusters located in the central opercular cortex and cerebellar lobule VIII and VIIB (S7 Table).

Sensitivity analyses showed very minimal effect of study site 1 on results, based on the calculation of the effect sizes for each region (S6 Table).

## Discussion

To our knowledge, this is the first study to investigate simultaneously the spontaneous brain activity (i.e., ALFF and fALFF) in offspring of parents with MDD and BD. In comparison to the control group, lower relative ALFF signals in HR-MDD and higher fALFF values in HR-BD were found at an FDR corrected threshold in the dorsal caudate nucleus and M1, respectively. At an uncorrected threshold, the HR-MDD group showed higher fALFF values in the central opercular cortex, compared to both control and HR-BD group. Both HR groups showed greater activity in the cerebellum than the control group.

Functional decoding of the dorsal caudate cluster, an FDR corrected cluster, highlighted a link with action which is coherent with the fact that the dorsal caudate nucleus is implicated in psychomotor functions and goal-directed action through dopaminergic mechanisms [59–61]. Cholinergic transmission is also thought to play a role in motor function since alteration of the VACht expression in caudate and putamen of patients with motor disorder has been noted [62]. Therefore, decreased activation in the dorsal caudate may be related to psychomotor dysfunction in MDD, which has been previously suggested [63]. Specifically, this neurobiological alteration of the caudate could explain the psychomotor retardation frequently seen in patients during a depressive episode [64]. Functional decoding also highlighted a role of the caudate cluster in motivation. While there is evident findings supporting the role of the ventral caudate in the brain reward system and motivation, the dorsal part appear more implicated in goal-directed behavior and motricity [65]. Globally, decreased activation of the caudate has been associated with higher level of anhedonia in patients with MDD [66]. This emotional disinterest could be associated with reduced goal-directed behavior. Therefore, decreased activation of this cluster at rest could be linked to higher anhedonia symptoms. D_1_ and D_2_ receptors were also associated with this cluster in our receptor/transporter analyses. As such, this is consistent with the fact that reduced striatal dopamine release has been associated with anhedonia in MDD [67]. Moreover, adjunctive treatment of depression with a partial D_2_ receptor agonist (e.g., aripiprazole) has shown promising results, especially for treatment-refractory MDD [68], which also suggests an implication of the dopamine system in depressive symptoms [67].

Because of its proximity and connections with the posterior insula and the postcentral gyrus [69,70], the central opercular cortex is implicated in interoceptive awareness [71], and in the processing of the affective component of pain [72]. This is consistent with the association of the central opercular with somatosensory properties observed in our functional decoding analysis such as physiological arousal. Altered connectivity between the posterior insula, postcentral gyrus and/or somatosensory regions has been previously highlighted in participants with MDD [73]. This dysfunction could result in greater physiological arousal which is known to be associated with increased emotion perception in MDD [74]. Increased physiological arousal has also been widely associated with higher level of anxiety symptoms [75]. In our study, groups did not differ in total score of anxiety, but a trend for higher score for the HR-MDD group was observed (p = 0.058). Therefore, this cluster could be associated with higher physiological arousal derived from more prevalent internalized symptoms in the HR-MDD group. Moreover, this cluster was also associated with the norepinephrine transporter (e.g., NET) [76]. Considering the role of the central opercular in pain perception and physiological arousal, the association observed with the NET is consistent with the key role of norepinephrine in stress management and physiological arousal [77]. In sum, higher fALFF signals in the central opercular cortex in HR-MDD participants could depict dysfunctional physiological response during resting-state.

Our second FDR corrected cluster was located in the M1. The M1 is implicated in action, action observation, and sensory information processing [78]. During mania, agitation and excessive movement are evident which has been interpreted as a failure to inhibit motor function [79]. Using a region-of-interest approach, hyperactivation of the M1 has been highlighted in participants with BD during reaction time task (motor performance) in 3 fMRI studies [80–82]. However, in two of these studies, both participants in a manic and depressive state displayed the same increased activation in the M1 in comparison to the control group. Therefore, alteration of the M1 could be state-independent, but more studies are warranted to examine this hypothesis. In our study, this cluster was found to be altered in both HR groups compared to the control group, but the effect survived the FDR correction only in the comparison between HR-BD and the control group. Focusing on MDD, a study highlighted decreased activation in M1 in participants with MDD and psychomotor retardation [83]. Hence, the preliminary evidence available suggests that BD is associated with M1 hyper-activation, while effects are mixed in MDD.

Our results showed that both HR groups showed abnormal activation of cerebellar lobule VI and of a cluster extending from lobule VIIII to a small portion of lobule VIIB. As such, our results are also consistent with the strong evidence of increased resting-state metabolism of the posterior cerebellum in both mood disorders [84,85], although the exact lobules impaired remain unclear. This is coherent with results of a meta-analysis of resting-state ALFF values that found alteration of the cerebellar lobule VI in both MDD and BD compared to controls [29], as well as a study which found that participants with MDD and first degree-relatives of patients with MDD displayed decreased regional homogeneity in the posterior cerebellum compared to the control group [86]. Cerebellar lobules VI, VIIB and VIII play crucial roles in motor skills, such as motor coordination, sensorimotor functions, and psychomotor functions, such as speed of processing [87,88]. In additional to these well-known roles, there is growing evidence of the implication of the lobule VI in several other functions, notably emotion processing (particularly positive emotions), working memory and (expressive) language [89]. As for lobules VIII and VIIB, growing evidence shows that these cerebellar regions are also involved in higher-level executive functions [90], such as set shifting and working memory [89]. Notably, most of these functions are altered in mood disorders [91–93] and in HR individuals (with effects of smaller magnitude) [94,95]. Therefore, alterations of these clusters in at-risk of mood disorder groups may reflect vulnerability to cognitive deficits, psychomotor deficits and/or emotional dysfunctions. However, the interpretation of the lobule VI result must be taken cautiously, since differences between groups were no longer significant after controlling for the number of negative life events and the severity of ADHD symptoms.

This study has some limitations. First, neuroimaging data was collected at 3 different sites, and this may have influenced results. However, we matched our groups on the study site to reduce the likelihood of this possibility. Secondly, the database is a community-referred cohort and thus, is heterogeneous in terms of children’s psychiatric diagnosis and symptomatology. Indeed, our groups presented gradient levels of anxiety and depressive symptoms, which were not significantly different between groups. While this lack of difference in symptoms may be explained by the heterogeneity of the original cohort, it must be considered that participants in each group were carefully matched in terms of study site, FSIQ, sex, and age. Alternatively, the lack of between-group difference in symptoms may be explained by the fact that youths involved in the current study were very young (e.g., mean age = 11.53 years), and it remains to be determined if the trends may become significant when they will become older. Finally, the FH-RDC does not have a perfect accuracy, which is a limitation to take into account when interpreting the between-group results. However, the FH-RDC is an instrument widely used in studies on the biological risk for mood disorders, due to its high specificity and tie very good inter-rater reliability [36].

## Conclusions

We found that offspring at risk of MDD displayed lower resting-state ALFF signals in an important region in psychomotor retardation and anhedonia, namely the dorsal caudate nucleus. This group also exhibited higher relative fALFF values in the central opercular, which may contribute to increased physiological arousal. Directly compared with controls, HR-BD participants showed increased fALFF values in an FDR corrected cluster located in the M1, which could be linked to agitation. Both HR groups showed alterations in the cerebellar which may be associated with the common vulnerability to mood disorders. Future studies should explore connectivity of regions found in this study to better characterized neurobiological deficits in HR and mood disorders. Further investigations of resting brain activity in young people at biological risk of mood disorders are needed to replicate the present findings and possibly identify biomarkers of these disorders before the conversion to a first episode. More importantly, longitudinal studies will need to investigate ALFF and fALFF alterations in individuals at biological risk for mood disorders and seek to determine if these alterations help predict those who transition to MDD or BD versus those who do not.

## Supporting information

S1 File Supplementary methods. fMRI Data acquisition.
(DOCX)

S1 Table Mean head motion and percentage of valid scans for included participants. Note. ALFF = amplitudes of low-frequency fluctuations; fALFF = fractioned amplitudes of low-frequency fluctuations; HR-MDD = high-risk of major depressive disorder; HR-BD = high risk of bipolar disorder; CTRL = control group.
(DOCX)

S2 Table Differential relative ALFF signals between participants at high-risk of major depressive disorder and participants at high-risk of bipolar disorder. Note. ALFF = amplitudes of low-frequency fluctuations; HR-MDD = high-risk of major depressive disorder; HR-BD = high risk of bipolar disorder; CTRL = control group; MNI = Montreal Neurological Institute; p_FDR_ = p-value corrected with false-discovery rate; *significant at an FDR corrected threshold.
(DOCX)

S3 Table Spatial Association with mental functions. Note. *(only results with a r≥0.30 are reported) ALFF = amplitudes of low-frequency fluctuations; fALFF = fractioned amplitudes of low-frequency fluctuations.
(DOCX)

S4 Table Spatial Association with receptor/transporter density maps*. Note. *(only results with a r≥0.30 are reported); ALFF = amplitudes of low-frequency fluctuations; fALFF = fractioned amplitudes of low-frequency fluctuations.
(DOCX)

S5 Table Analyses of variance and between-group comparisons with and without Bonferonni corrections for covariates. Note. *Significant at a threshold of p<0.01 corrected with Bonferroni. ALFF = amplitudes of low-frequency fluctuations; fALFF = fractioned amplitudes of low-frequency fluctuations; HR-MDD = high-risk of major depressive disorder; HR-BD = high risk of bipolar disorder; CTRL = control group; L = left; R = Right; NLES-P = Negative Life Events Scale–Parent Report; SWAN = Strengths and Weaknesses Assessment for ADHD and Normal Behavior; MNI = Montreal Neurological Institute; MFQ = Mood and Feelings Questionnaire; SCARED = Screen for Child Anxiety Related Disorders.
(DOCX)

S6 Table Sensitivity analyses: effect size estimates (Cohen’s d) for each comparison. Note. ALFF = amplitudes of low frequency fluctuations; fALFF = fractioned amplitudes of low frequency fluctuations; HR-MDD = high-risk of major depressive disorder; HR-BD = high risk of bipolar disorder.
(DOCX)

S7 Table Differential relative fALFF signals between participants at high-risk of major depressive disorder and participants at high-risk of bipolar disorder. Note. fALFF = fractioned amplitudes of low-frequency fluctuations; HR-MDD = high-risk of major depressive disorder; HR-BD = high risk of bipolar disorder; CTRL = control group; MNI = Montreal Neurological Institute; p_FDR_ = p-value corrected with false-discovery rate; *significant at an FDR corrected threshold.
(DOCX)
