# Supplementary material for: Altered resting-state amplitudes of low-frequency fluctuations in offspring of parents with a diagnosis of bipolar disorder or major depressive disorder
Source: PLoS One. 2025 Feb 18;20(2):e0316330. doi: 10.1371/journal.pone.0316330 (PMC11835319; doi:10.1371/journal.pone.0316330)
Supplement: S1 File — fMRI Data acquisition. (DOCX) [file pone.0316330.s001.docx]

**fMRI Data acquisition**

Echo-planar imaging sequences for functional runs at the mobile site in Staten Island used TR=1450, TE=40, FA=55°, slice thickness=2.5mm, number of slices=54, FOV=192mm, a multiband factor of 3, and voxel size=2.5x2.5x2.5mm. Both RUBIC and CBIC used the same data acquisition protocol which consisted of two resting-state scans of 5 min each with TR=800ms, TE=30ms, FA=31°, slice thickness=2.4mm, number of slices=60, FOV=204mm, a multiband factor of 6, and voxel size=2.4x2.4x2.4mm (more details on acquisition protocols and parameters can be found in (Alexander et al., 2017)^[[1]](#footnote-1)^ and at <http://fcon_1000.projects.nitrc.org/indi/cmi_healthy_brain_network/index.html>).

1. Alexander, L.M., Escalera, J., Ai, L., Andreotti, C., Febre, K., Mangone, A., Vega-Potler, N., Langer, N., Alexander, A., Kovacs, M., Litke, S., O’Hagan, B., Andersen, J., Bronstein, B., Bui, A., Bushey, M., Butler, H., Castagna, V., Camacho, N., Chan, E., Citera, D., Clucas, J., Cohen, S., Dufek, S., Eaves, M., Fradera, B., Gardner, J., Grant-Villegas, N., Green, G., Gregory, C., Hart, E., Harris, S., Horton, M., Kahn, D., Kabotyanski, K., Karmel, B., Kelly, S.P., Kleinman, K., Koo, B., Kramer, E., Lennon, E., Lord, C., Mantello, G., Margolis, A., Merikangas, K.R., Milham, J., Minniti, G., Neuhaus, R., Levine, A., Osman, Y., Parra, L.C., Pugh, K.R., Racanello, A., Restrepo, A., Saltzman, T., Septimus, B., Tobe, R., Waltz, R., Williams, A., Yeo, A., Castellanos, F.X., Klein, A., Paus, T., Leventhal, B.L., Craddock, R.C., Koplewicz, H.S., Milham, M.P., 2017. An open resource for transdiagnostic research in pediatric mental health and learning disorders. Sci. Data 4, 170181. https://doi.org/10.1038/sdata.2017.181 [↑](#footnote-ref-1)
