# Supplementary material for: Altered resting-state amplitudes of low-frequency fluctuations in offspring of parents with a diagnosis of bipolar disorder or major depressive disorder
Source: PLoS One. 2025 Feb 18;20(2):e0316330. doi: 10.1371/journal.pone.0316330 (PMC11835319; doi:10.1371/journal.pone.0316330)
Supplement: S1 Table — Note. ALFF = amplitudes of low-frequency fluctuations; fALFF = fractioned amplitudes of low-frequency fluctuations; HR-MDD = high-risk of major depressive disorder; HR-BD = high risk of bipolar disorder; CTRL = control group. (DOCX) [file pone.0316330.s002.docx]

| Table S1. Mean head motion and percentage of valid scans for included participants. | | | | | |
| --- | --- | --- | --- | --- | --- |
| ALFF | | | | | |
|  | **HR-MDD (n=150)** | **HR-BD (n=50)** | **CTRL (n=150)** | **F-value** | **p-value** |
|  | Mean (sd) | | |  |  |
| Mean motion | 0.280 (0.395) | 0.226 (0.173) | 0.316 (0.435) | 0.998 | 0.370 |
| Percentage valid scans | 88.26% (15.71%) | 90.98% (11.21%) | 87.82% (17.17%) | 0.768 | 0.465 |
| fALFF | | | | | |
|  | **HR-MDD (n=149)** | **HR-BD (n=50)** | **CTRL (n=150)** | **Statistic** | **p-value** |
|  | Mean (sd) | | |  |  |
| Mean motion | 0.296 (0.396) | 0.226 (0.173) | 0.316 (0.435) | 0.996 | 0.370 |
| Percentage valid scans | 88.22% (15.75%) | 90.98% (11.21%) | 87.82% (17.17%) | 0.773 | 0.463 |
| Note. ALFF = amplitudes of low-frequency fluctuations; fALFF = fractioned amplitudes of low-frequency fluctuations; HR-MDD = high-risk of major depressive disorder; HR-BD = high risk of bipolar disorder; CTRL = control group | | | | | |
