# Supplementary material for: Altered resting-state amplitudes of low-frequency fluctuations in offspring of parents with a diagnosis of bipolar disorder or major depressive disorder
Source: PLoS One. 2025 Feb 18;20(2):e0316330. doi: 10.1371/journal.pone.0316330 (PMC11835319; doi:10.1371/journal.pone.0316330)
Supplement: S2 Table — Note. ALFF = amplitudes of low-frequency fluctuations; HR-MDD = high-risk of major depressive disorder; HR-BD = high risk of bipolar disorder; CTRL = control group; MNI = Montreal Neurological Institute; pFDR = p-value corrected with false-discovery rate; *significant at an FDR corrected threshold. (DOCX) [file pone.0316330.s003.docx]

| Table S2. Differential relative ALFF signals between participants at high-risk of major depressive disorder and participants at high-risk of bipolar disorder | | | | | | | | |
| --- | --- | --- | --- | --- | --- | --- | --- | --- |
| Contrast | | **L/R** | **Regions** | **MNI coordinates (x,y,z)** | **Voxels** | **F or T value** | **p-FDR corrected (cluster-level)** | **p-value uncorrected**  **(peak-level)** |
|  |  |  |  |  |  |  |  |  |
| HR-MDD > HR-BD | | | |  |  |  |  |  |
|  | | | None |  |  |  |  |  |
| HR-BD > HR-MDD | | | |  |  |  |  |  |
|  | | | None |  |  |  |  |  |
| CTRL > HR-MDD | | |  |  |  |  |  |  |
|  | R | | Posterior midcingulate cortex | 16,-8,46 | 22 | 4.42 | 0.767 | <0.001 |
|  | L | | Dorsal cingulum bundle | -10,8,24 | 58 | 4.28 | 0.384 | <0.001 |
|  | R | | Midcingulate cortex | 14,14,44  16,6,48 | 33 | 4.27 | 0.767 | <0.001 |
|  | R | | Caudate | 16,16,22  16,8,20  18,-10,30 | 176 | 4.17 | **0.010*** | <0.001 |
| HR-MDD > CTRL | | |  |  |  |  |  |  |
|  | R | | Cerebellar lobule VIII and VIIB | 34,-60,-44  30,-52,-44  40,-54,-44 | 45 | 4.24 | 0.765 | <0.001 |
|  | L | | Inferior frontal gyrus | -48,16,-4 | 20 | 4.01 | 0.767 | <0.001 |
|  | L | | Central opercular | -60,-18,28 | 20 | 3.82 | 0.767 | <0.001 |
| CTRL > HR-BD | | |  |  |  |  |  |  |
|  |  | | None |  |  |  |  |  |
| HR-BD > CTRL | | |  |  |  |  |  |  |
|  | R | | Cerebellar, lobule VI | 34,-42,-30  28,-36,-28 | 32 | 4.34 | 0.275 | <0.001 |
|  | L | | M1 | -34,-22,46 | 38 | 3.80 | 0.275 | <0.001 |
| Note. ALFF = amplitudes of low-frequency fluctuations; HR-MDD = high-risk of major depressive disorder; HR-BD = high risk of bipolar disorder; CTRL = control group; MNI = Montreal Neurological Institute; p_FDR_ = p-value corrected with false-discovery rate; *significant at an FDR corrected threshold | | | | | | | | |
