# Supplementary material for: Altered resting-state amplitudes of low-frequency fluctuations in offspring of parents with a diagnosis of bipolar disorder or major depressive disorder
Source: PLoS One. 2025 Feb 18;20(2):e0316330. doi: 10.1371/journal.pone.0316330 (PMC11835319; doi:10.1371/journal.pone.0316330)
Supplement: S3 Table — Note. *(only results with a r≥0.30 are reported) ALFF = amplitudes of low-frequency fluctuations; fALFF = fractioned amplitudes of low-frequency fluctuations. (DOCX) [file pone.0316330.s004.docx]

| Table S3. Spatial Association with mental functions | |
| --- | --- |
| Mental functions | **Mean Fisher's z (Spearman rho)** |
| *ALFF Dorsal caudate nucleus (16,8,18)* | |
| Motivation | 0.5208 |
| Cognitive control | 0.5200 |
| Multi demand | 0.5023 |
| Physiological arousal | 0.4192 |
| Language | 0.3606 |
| *fALFF Central opercular cortex (-60,-16,28)* | |
| Physiological arousal | 0.5677 |
| Action | 0.5083 |
| Cognitive control | 0.4440 |
| Multi demand | 0.4307 |
| Language | 0.3658 |
| Auditory perception | 0.3569 |
| Note. *(only results with a r>0.30 are reported) ALFF = amplitudes of low-frequency fluctuations; fALFF = fractioned amplitudes of low-frequency fluctuations | |
