# Supplementary material for: Altered resting-state amplitudes of low-frequency fluctuations in offspring of parents with a diagnosis of bipolar disorder or major depressive disorder
Source: PLoS One. 2025 Feb 18;20(2):e0316330. doi: 10.1371/journal.pone.0316330 (PMC11835319; doi:10.1371/journal.pone.0316330)
Supplement: S4 Table — Note. *(only results with a r≥0.30 are reported); ALFF = amplitudes of low-frequency fluctuations; fALFF = fractioned amplitudes of low-frequency fluctuations. (DOCX) [file pone.0316330.s005.docx]

| Table S4. Spatial Association with receptor/transporter density maps* | |
| --- | --- |
| Receptor/transporter density maps | **Mean Fisher's z**  **(Spearman rho)** |
| *ALFF Dorsal caudate nucleus (16,8,18)* | |
| D2 | 0.5062 |
| D1 | 0.5061 |
| VAChT | 0.4693 |
| DAT | 0.4602 |
| MOR | 0.4435 |
| 5HT6 | 0.4252 |
| H3 | 0.4215 |
| 5HTT | 0.3729 |
| NMDA | 0.3209 |
| 5HT1B | 0.3205 |
| mGluR5 | 0.3193 |
| 5HT4 | 0.3087 |
| *fALFF Central opercular cortex (-60,-16,28)* | |
| mGluR5 | 0.4069 |
| 5HT1B | 0.4039 |
| NAT | 0.3928 |
| 5HT6 | 0.3904 |
| CB1 | 0.3775 |
| MOR | 0.3629 |
| H3 | 0.3247 |
| M1 | 0.3217 |
| 5HT2A | 0.3192 |
| GABA | 0.3119 |
| NMDA | 0.3091 |
| D1 | 0.3058 |
| Note. *(only results with a r>0.30 are reported); ALFF = amplitudes of low-frequency fluctuations; fALFF = fractioned amplitudes of low-frequency fluctuations | |
