# Supplementary material for: Altered resting-state amplitudes of low-frequency fluctuations in offspring of parents with a diagnosis of bipolar disorder or major depressive disorder
Source: PLoS One. 2025 Feb 18;20(2):e0316330. doi: 10.1371/journal.pone.0316330 (PMC11835319; doi:10.1371/journal.pone.0316330)
Supplement: S5 Table — Note. *Significant at a threshold of p<0.01 corrected with Bonferroni. ALFF = amplitudes of low-frequency fluctuations; fALFF = fractioned amplitudes of low-frequency fluctuations; HR-MDD = high-risk of major depressive disorder; HR-BD = high risk of bipolar disorder; CTRL = control group; L = left; R = Right; NLES-P = Negative Life Events Scale–Parent Report; SWAN = Strengths and Weaknesses Assessment for ADHD and Normal Behavior; MNI = Montreal Neurological Institute; MFQ = Mood and Feelings Questionnaire; SCARED = Screen for Child Anxiety Related Disorders. (DOCX) [file pone.0316330.s006.docx]

| Table S5. Analyses of variance and between-group comparisons with and without Bonferonni corrections for covariates. | | | | | | | | | | | | | |
| --- | --- | --- | --- | --- | --- | --- | --- | --- | --- | --- | --- | --- | --- |
| Contrast | Regions | MNI coordinates (x,y,z) | Comparison | Anova | | Ancova (NLES-P number of events) | | Ancova (SWAN total score) | | Ancova (MFQ total score) | | Ancova (SCARED total score) | |
|  |  |  |  | F-value | p-value | F-value | p-value | F-value | p-value | F-value | p-value | F-value | p-value |
| *ALFF* | | | | | | | | | | | | | |
| ANY EFFECT | R dorsal caudate nucleus | 16,8,18 | CTRL > HR-MDD | 11.036 | <0.001* | 6.361 | <0.001* | 8.138 | <0.001* | 6.452 | <0.001* | 7.710 | <0.001* |
|  |  |  | HR-BD > HR-MDD |  | 0.017 |  | 0.024 |  | 0.015 |  | 0.039 |  | 0.021 |
|  |  |  | HR-BD vs CTRL |  | 1.000 |  | 1.000 |  | 1.000 |  | 1.000 |  | 1.000 |
| HR > CTRL | R cerebellar, lobule VI | 34,-44,-30 | HR-MDD > CTRL | 8.906 | 0.044 | 5.351 | 0.047 | 5.648 | 0.095 | 6.038 | 0.026 | 6.441 | 0.128 |
|  |  |  | HR-BD > CTRL |  | <0.001* |  | <0.001* |  | <0.001* |  | <0.001* |  | <0.001* |
|  | R cerebellar, lobule VIII & VIIB | 34,-60,-44 | HR-MDD > CTRL | 9.215 | <0.001* | 4.362 | 0.020 | 5.199 | 0.003* | 4.713 | 0.005* | 5.034 | 0.008* |
|  |  |  | HR-BD > CTRL |  | 0.004* |  | 0.017 |  | 0.012 |  | 0.009* |  | 0.011 |
|  | L primary motor cortex | -38,-18,56 | HR-MDD > CTRL | 8.577 | 0.036 | 5.992 | 0.147 | 5.419 | 0.173 | 5.584 | 0.021 | 6.211 | 0.033 |
|  |  |  | HR-BD > CTRL |  | <0.001* |  | <0.001* |  | 0.002* |  | <0.001* |  | <0.001* |
| *fALFF* | | | | | | | | | | | | | |
| ANY EFFECT | L central opercular cortex | -60,-16,28 | HR-MDD > CTRL | 11.312 | <0.001* | 6.256 | <0.001* | 9.354 | <0.001* | 5.011 | 0.001* | 6.210 | <0.001* |
|  |  |  | HR- MDD > HR- BD |  | 0.004* |  | 0.009* |  | 0.005* |  | 0.052 |  | 0.010* |
|  |  |  | HR-BD vs CTRL |  | 1.000 |  | 1.000 |  | 1.000 |  | 1.000 |  | 1.000 |
| HR > CTRL | R cerebellar lobule VIII & VIIB | 34,-62,-44 | HR-MDD > CTRL | 13.295 | <0.001* | 8.020 | <0.001* | 8.058 | <0.001* | 6.022 | 0.004* | 7.062 | 0.003* |
|  |  |  | HR-BD > CTRL |  | <0.001* |  | <0.001* |  | <0.001* |  | <0.001* |  | 0.001* |
|  | L primary motor cortex | -38,-18,54 | HR-MDD > CTRL | 8.331 | 0.083 | 6.186 | 0.241 | 5.928 | 0.284 | 6.102 | 0.023 | 6.386 | 0.047 |
|  |  |  | HR-BD > CTRL |  | <0.001* |  | <0.001* |  | 0.002* |  | <0.001* |  | <0.001* |
| Note. *Significant at a threshold of p<0.01 corrected with Bonferroni. ALFF = amplitudes of low-frequency fluctuations; fALFF = fractioned amplitudes of low-frequency fluctuations; HR-MDD = high-risk of major depressive disorder; HR-BD = high risk of bipolar disorder; CTRL = control group; L = left; R = Right; NLES-P = Negative Life Events Scale – Parent Report; SWAN = Strengths and Weaknesses Assessment for ADHD and Normal Behavior; MNI = Montreal Neurological Institute; MFQ = Mood and Feelings Questionnaire; SCARED = Screen for Child Anxiety Related Disorders. | | | | | | | | | | | | | |
