# Supplementary material for: Altered resting-state amplitudes of low-frequency fluctuations in offspring of parents with a diagnosis of bipolar disorder or major depressive disorder
Source: PLoS One. 2025 Feb 18;20(2):e0316330. doi: 10.1371/journal.pone.0316330 (PMC11835319; doi:10.1371/journal.pone.0316330)
Supplement: S6 Table — Note. ALFF = amplitudes of low frequency fluctuations; fALFF = fractioned amplitudes of low frequency fluctuations; HR-MDD = high-risk of major depressive disorder; HR-BD = high risk of bipolar disorder. (DOCX) [file pone.0316330.s007.docx]

| Table S6. Sensitivity analyses: effect size estimates (Cohen’s d) for each comparison | | |
| --- | --- | --- |
|  | With study site 1 | Without study site 1 |
| ALFF – dorsal caudate nucleus | | |
| Controls vs HR-MDD | 0.341 | 0.348 |
| Controls vs HR-BD | 0.344 | 0.350 |
| HR-MDD vs HR-BD | 0.349 | 0.352 |
| ALFF – Cerebellar lobule VI | | |
| High risk vs controls | 0.419 | 0.432 |
| ALFF – Cerebellar lobule VIII and VIIB | | |
| High risk vs controls | 0.531 | 0.530 |
| ALFF – primary motor cortex | | |
| High risk vs controls | 0.603 | 0.604 |
| fALFF – central opercular cortex | | |
| Controls vs HR-MDD | 0.551 | 0.553 |
| Controls vs HR-BD | 0.529 | 0.525 |
| HR-MDD vs HR-BD | 0.519 | 0.533 |
| fALFF – Cerebellar lobule VIII and VIIB | | |
| High risk vs controls | 0.405 | 0.406 |
| fALFF – primary motor cortex | | |
| High risk vs controls | 0.681 | 0.685 |
| Note. ALFF = amplitudes of low frequency fluctuations; fALFF = fractioned amplitudes of low frequency fluctuations; HR-MDD = high-risk of major depressive disorder; HR-BD = high risk of bipolar disorder. | | |
