# Supplementary material for: Altered resting-state amplitudes of low-frequency fluctuations in offspring of parents with a diagnosis of bipolar disorder or major depressive disorder
Source: PLoS One. 2025 Feb 18;20(2):e0316330. doi: 10.1371/journal.pone.0316330 (PMC11835319; doi:10.1371/journal.pone.0316330)
Supplement: S7 Table — Note. fALFF = fractioned amplitudes of low-frequency fluctuations; HR-MDD = high-risk of major depressive disorder; HR-BD = high risk of bipolar disorder; CTRL = control group; MNI = Montreal Neurological Institute; pFDR = p-value corrected with false-discovery rate; *significant at an FDR corrected threshold. (DOCX) [file pone.0316330.s008.docx]

| Table S7. Differential relative fALFF signals between participants at high-risk of major depressive disorder and participants at high-risk of bipolar disorder | | | | | | | |
| --- | --- | --- | --- | --- | --- | --- | --- |
| Contrast | **L/R** | **Regions** | **MNI coordinates (x,y,z)** | **Voxels** | **F or T value** | **p-FDR corrected (cluster-level)** | **p-value uncorrected**  **(peak-level)** |
|  |  |  |  |  |  |  |  |
| HR-MDD > HR-BD | | |  |  |  |  |  |
|  | L | Inferior frontal gyrus | -46,22,-4 | 30 | 3.77 | 0.357 | <0.001 |
| HR-BD > HR-MDD | | |  |  |  |  |  |
|  | R | Lateral orbitofrontal cortex | 32,22,-20  30,24,-28 | 30 | 3.73 | 0.601 | <0.001 |
| CTRL > HR-MDD |  |  |  |  |  |  |  |
|  |  | None |  |  |  |  |  |
| HR-MDD > CTRL |  |  |  |  |  |  |  |
|  | L | Central opercular | -60,-16,28 | 45 | 4.36 | 0.240 | <0.001 |
|  | R | Cerebellar, lobule 8 and 7B | 34,-62,-44 | 50 | 3.90 | 0.662 | <0.001 |
| CTRL > HR-BD |  |  |  |  |  |  |  |
|  |  | None |  |  |  |  |  |
| HR-BD > CTRL |  |  |  |  |  |  |  |
|  | L | M1 | -38,-18,52  -34,-22,46 | 60 | 4.21 | **0.042*** | <0.001 |
|  | R | Cerebellar, lobule 6 | 34,-38,-28 | 20 | 3.58 | 0.409 | <0.001 |
| Note. fALFF = fractioned amplitudes of low-frequency fluctuations; HR-MDD = high-risk of major depressive disorder; HR-BD = high risk of bipolar disorder; CTRL = control group; MNI = Montreal Neurological Institute; p_FDR_ = p-value corrected with false-discovery rate; *significant at an FDR corrected threshold | | | | | | | |
